# Modeling Disease Severity in Multiple Sclerosis Using Electronic Health Records

**DOI:** 10.1371/journal.pone.0078927

**Published:** 2013-11-11

**Authors:** Zongqi Xia, Elizabeth Secor, Lori B. Chibnik, Riley M. Bove, Suchun Cheng, Tanuja Chitnis, Andrew Cagan, Vivian S. Gainer, Pei J. Chen, Katherine P. Liao, Stanley Y. Shaw, Ashwin N. Ananthakrishnan, Peter Szolovits, Howard L. Weiner, Elizabeth W. Karlson, Shawn N. Murphy, Guergana K. Savova, Tianxi Cai, Susanne E. Churchill, Robert M. Plenge, Isaac S. Kohane, Philip L. De Jager

**Affiliations:** 1 Department of Neurology, Brigham and Women’s Hospital, Boston, Massachusetts, United States of America; 2 Harvard Medical School, Boston, Massachusetts, United States of America; 3 Program in Medical and Population Genetics, Broad Institute, Cambridge, Massachusetts, United States of America; 4 Department of Biostatistics, Dana-Farber Cancer Institute, Boston, Massachusetts, United States of America; 5 Research Computing and Informatics Service, Partners HealthCare, Charlestown, Massachusetts, United States of America; 6 Department of Pediatrics, Boston Children’s Hospital, Boston, Massachusetts, United States of America; 7 Department of Medicine, Brigham and Women’s Hospital, Boston, Massachusetts, United States of America; 8 Center for System Biology, Massachusetts General Hospital, Boston, Massachusetts, United States of America; 9 Department of Medicine, Massachusetts General Hospital, Boston, Massachusetts, United States of America; 10 Laboratory for Computer Science, Massachusetts Institute of Technology, Cambridge, Massachusetts, United States of America; 11 Laboratory of Computer Science, Massachusetts General Hospital, Charlestown, Massachusetts, United States of America; 12 Department of Biostatistics, Harvard School of Public Health, Boston, Massachusetts, United States of America; 13 i2b2/National Center for Biomedical Computing, Partners HealthCare, Boston, Massachusetts, United States of America; University of Maryland, College Park, United States of America

## Abstract

**Objective:**

To optimally leverage the scalability and unique features of the electronic health records (EHR) for research that would ultimately improve patient care, we need to accurately identify patients and extract clinically meaningful measures. Using multiple sclerosis (MS) as a proof of principle, we showcased how to leverage routinely collected EHR data to identify patients with a complex neurological disorder and derive an important surrogate measure of disease severity heretofore only available in research settings.

**Methods:**

In a cross-sectional observational study, 5,495 MS patients were identified from the EHR systems of two major referral hospitals using an algorithm that includes codified and narrative information extracted using natural language processing. In the subset of patients who receive neurological care at a MS Center where disease measures have been collected, we used routinely collected EHR data to extract two aggregate indicators of MS severity of clinical relevance multiple sclerosis severity score (MSSS) and brain parenchymal fraction (BPF, a measure of whole brain volume).

**Results:**

The EHR algorithm that identifies MS patients has an area under the curve of 0.958, 83% sensitivity, 92% positive predictive value, and 89% negative predictive value when a 95% specificity threshold is used. The correlation between EHR-derived and true MSSS has a mean R^2^ = 0.38±0.05, and that between EHR-derived and true BPF has a mean R^2^ = 0.22±0.08. To illustrate its clinical relevance, derived MSSS captures the expected difference in disease severity between relapsing-remitting and progressive MS patients after adjusting for sex, age of symptom onset and disease duration (p = 1.56×10^−12^).

**Conclusion:**

Incorporation of sophisticated codified and narrative EHR data accurately identifies MS patients and provides estimation of a well-accepted indicator of MS severity that is widely used in research settings but not part of the routine medical records. Similar approaches could be applied to other complex neurological disorders.

## Introduction

With the increasing integration of electronic health records (EHR) into routine clinical care, there is an emerging interest in harnessing the wealth of EHR data for clinical research that ultimately improve patient care. Optimal use of EHR data for clinical research that would ultimately improve patient outcomes requires efficient extraction of meaningful information from codified data (e.g., demographics, billing codes for diagnoses and procedures, laboratory results, electronic prescriptions) and narrative data (e.g., clinical encounter notes, imaging reports) to accurately identify patient cohorts and measure clinically relevant outcomes [1], [2]. The prevailing approach that relies exclusively on administrative billing codes can be limited in accuracy and may miss relevant phenotypes [3]. The growing availability and functionality of the EHR system together with advances in natural language processing (NLP) and bioinformatics methods that are essential for extracting meaningful clinical information from the EHR data have converged to enable efficient and cost-effective development of EHR-derived patient cohorts and large-scale assessment of phenotypes relevant to patient care [1], [2], [4]. Our group has built a framework [5], [6], [7] to successfully leverage EHR for research in diseases such as asthma [8], depression [9], inflammatory bowel disease [10], and rheumatoid arthritis [11], [12], [13], [14]. In parallel, important work led by the Electronic Medical Records and Genomics (eMERGE) Network has further demonstrated the broad potential of EHR-based approaches in discovery and clinical research [15], [16], [17].

Neurological research leveraging the EHR data is just emerging [15], in part because the complexity of neurological diseases creates challenges in deriving relevant disease outcomes not available from routine clinical encounters. Using multiple sclerosis (MS) as a proof of principle, we set out to develop a potentially generalizable informatics approach that would enable EHR research in neurological diseases. MS typically consists of a relapsing-remitting inflammatory phase and, in many patients, an underlying progressive neurodegenerative course that makes this demyelinating disease of the central nervous system a leading cause of neurological disability in younger adults [18]. One of the best predictors of long-term neurological disability in MS is atrophy on brain magnetic resonance imaging (MRI) [19].

Given that MS patients display heterogeneity in their disease course and the increasing options for MS treatment have reduced the number of patients on any specific medication, sample size is limited for conducting patient-oriented research in MS such as pharmaco-epidemiology or pharmacogenomic studies. Outcome data traditionally come from well-designed prospective cohorts, including the Comprehensive Longitudinal Investigation of MS at Brigham and Women’s Hospital (CLIMB) [20], which provided critical data for this study. While EHR-derived cohorts will not replace clinic or population-based studies, they capture a larger number of patients and provide unique and complementary features not found in traditional cohorts. Clinically relevant outcomes not easily attainable from routine medical records are crucial for research in EHR-derived cohorts. Here, we report a rigorous EHR-based informatics approach to (1) accurately identify MS patients and (2) provide a surrogate measure of disease severity in MS patients. Our study makes the first step towards realizing a major goal in personalized medicine: leveraging each individual’s unique, routinely collected health information to inform clinical outcome and improve patient care.

## Methods

### Electronic Health Records Source

The Institutional Review Board of Partners HealthCare approved all aspects of this study, including the waiver of written consent for use of de-identified EHR data for research. The Partners HealthCare EHR system captures nearly five million patients and contains over one billion clinical observations dating back to 1994 for the Massachusetts General Hospital and 1996 for the Brigham and Women’s Hospital, both of which are Harvard-affiliated teaching hospitals and major tertiary care centers in the New England area.

### EHR Algorithms for Classifying MS

**Figure 1:**
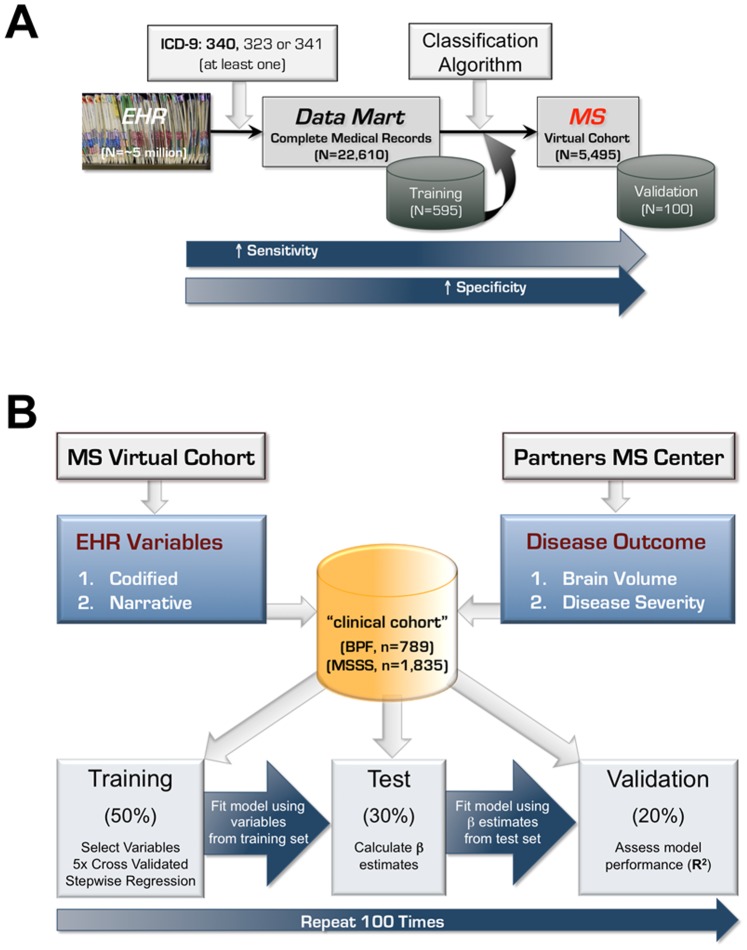
Overall approach for developing EHR algorithm to classify multiple sclerosis (A), and to derive surrogate measures of brain parenchymal fraction and multiple sclerosis severity score in MS patients (B).

From the Partners HealthCare EHR system, we developed an EHR-based classification algorithm to identify MS patients (see **Figure 1A** for summary of the overall approach). As a first step, we included any patient with at least one MS-related International Classification of Disease 9^th^ edition (ICD-9) code (340, 323 or 341). Using these 22,610 patients, we created an “MS data mart” containing the complete medical records (as of February 2011) of all their visits to Partners HealthCare sites. Similar to our prior efforts to identify patients with rheumatoid arthritis and inflammatory bowel disease from the EHR system [10], [13], we generated a list of clinician expert-defined, MS-relevant codified and narrative variables from the EHR data for each patient. Variables are excluded if the frequency of occurrence in the data mart was 10% or less.

Codified variables were derived from billing codes for diagnoses and procedures, demographic information and electronic prescriptions. For codified variables, we counted the number of occurrences per patient (e.g., ICD-9 code for MS, procedure code for MRI of the brain or cervical spine or orbit, electronic prescriptions for any of the MS disease-modifying medications). We also included derivatives of billing codes (e.g., annualized ICD-9 code for MS, proportion of the total ICD-9 codes in the EHR belonging to MS).

Narrative variables on symptoms, signs, medications, MRI reports, and neurologist’s impression and treatment plan were extracted from free-text clinical narratives (outpatient notes, discharge summaries, imaging reports and pathology reports) using the clinical Text Analytics and Knowledge Extraction System (cTAKES) NLP system (ctakes.apache.org) [21], which parses the texts to identify clinically relevant concepts and the associated qualifying attributes (negated, non-negated). Two neurologists with subspecialty expertise in MS and neuroimmunology created a customized dictionary of MS-relevant terms based on their clinical experiences and further refined the dictionary after reviewing 60 randomly selected clinical notes or imaging reports from known MS patients that were annotated by cTAKES. Additional MS neurologists reviewed the list of narrative terms and provided feedback. The refined list of expert-defined terms were mapped to two health care terminology indices to allow for language variations: (1) Systematized Nomenclature of Medicine Clinical Terms (SNOMED-CT) (http://www.nlm.nih.gov/research/umls/Snomed/snomed_main.html) serves to organize terms for signs and symptoms, anatomical sites, disease terms and procedures; (2) RxNorm (http://www.nlm.nih.gov/research/umls/rxnorm/) serves to organize terms for generic and brand name medications. For each narrative variable, we determined the sum of positive and negative mentions per patient.

Based on an estimation of the number of subjects needed to develop a classification algorithm, 595 patients from the MS data mart were randomly selected for a training set to develop the MS-classifying algorithm. One neurologist reviewed the medical records of all patients in the training set to establish whether a patient had a definitive diagnosis of MS, which was supported by documentation in a neurologist’s clinical note or a relevant MRI report. We fit LASSO penalized logistic regression models with Bayesian Information Criterion [22] to select informative EHR variables for predicting MS diagnosis and estimated their regression parameters.

To assess the performance of these MS-classifying models, we calculated the area under the curve (AUC) using a receiver operating characteristic analysis as well as sensitivity, positive predictive value (PPV), and negative predictive value (NPV) at 95% specificity. To correct for overfitting bias, the 0.632 bootstrap cross-validation was used to obtain bias corrected estimates of all performance parameters [23]. The standard error estimates were obtained using a bootstrap procedure with 1,000 replicates. For all models, we selected a probability threshold corresponding to 95% specificity and classified patients with a probability exceeding the threshold value as having a definitive diagnosis of MS. The best algorithm was then applied to all 22,610 patients in the MS data mart to assign a probability of definitive MS for each patient and established an EHR-based cohort of MS patients. From this cohort, two neurologists reviewed the medical records of 100 randomly selected patients for independent validation of MS diagnosis.

### Multiple Sclerosis Center Patients

A subset of the EHR-derived MS cohort receives neurological care at the Partners Multiple Sclerosis Center, including patients who are enrolled in CLIMB, an ongoing prospective natural-history cohort study [20]. To develop EHR-based algorithms for brain volume and MS severity, we used the subset of MS patients with existing brain MRI and clinical outcomes collected prospectively and available from the MS Center database. These outcomes are collected separately from the EHR data. All patients met the revised McDonald diagnostic criteria for MS [24]. We established an interface between the EHR system and the MS Center database to access data that are not available from routine medical records, including manually corrected brain parenchymal fraction (BPF), the multiple sclerosis severity score (MSSS), and MS disease category (relapsing-remitting, RR; secondary progressive, SP; primary progressive, PP). Disease outcome data are based on values collected at each patient’s last available visit as of August 2012. The supplemental text contains details on the neuroimaging approach and MSSS measure.

Neuroimaging approach has been described in detail elsewhere [25], [26], [27]. Briefly, dual-echo proton density and T2-weighted axial images of 3-mm thick sections from routine clinical brain MRI scans on a 1.5 Tesla system were segmented using a semi-automated pipeline. MRI scans underwent quality control with manual correction of detected tissue misclassification to provide corrected BPF. For each patient, only the corrected BPF measure from the most recent brain MRI scan was used for algorithm development.

MSSS is a method for quantifying MS disease severity in the course of MS, based on a single assessment of a clinical indicator of MS disability known as the Kurtzke Expanded Disability Status Scale (EDSS) score, adjusted for disease duration [28]. Disease duration is defined as the time interval from the self-reported symptom onset to the recorded clinic encounter. Using EDSS and disease duration from the most recent clinic visit, we calculated the MSSS for each patient. We excluded any EDSS measures captured within the first two years of symptom onset, given that MSSS is not robust at this stage of the disease.

### EHR Algorithms for Deriving Brain Volume and Disease Severity in MS

Using a list of expert-defined EHR variables (**Table S1**) and existing brain MRI and clinical features, we develop algorithms for brain volume and MS disease severity. We included all patients with available BPF and MSSS after 2004 to maximize sample size. To reduce the confounding effects of race and ethnicity, which influence disease course [29], we only included patients with self-reported European ancestry (representing approximately 90% of the patients in the MS Center, n = 789 for BPF, n = 1,835 for MSSS).

To develop EHR algorithms for BPF and MSSS, we took a double cross-validation approach (**Figure 1B**). Subjects are divided into a training (50%), test (30%) and validation (20%) subgroup. In the *training* set, we performed a 5-fold cross-validated stepwise regression to select EHR variables to be included in the algorithms. To avoid over-fitting the algorithms in the training set, we determined, in the independent *test* set, the magnitude of the beta coefficient (or the weight) of each EHR variable selected from the training set, to create the final algorithms. We applied the algorithms to the independent *validation* set to assess algorithm performance as measured by the correlation between algorithm-derived and corresponding known outcome (either BPF or MSSS) using R^2^ adjusted for the number of variables. The entire process, starting with dividing the subjects into test, training and validation set, was repeated 100 times to obtain the mean R^2^ for the BPF and MSSS algorithms.

EHR variables that had a non-zero value in <10% subjects were removed. In addition to EHR variables, we evaluated the algorithm performance when considering the following variables that are obtained from the MS Center database: age at first symptom, sex and disease duration for BPF, and age at first symptom and sex for MSSS. The details of determining the optimal frequency threshold for the EHR variables are described in the text S1 (**Table S2**, **Figure S3**).

## Results

### Establishing EHR-based MS Cohort

Our approach to identify an EHR-derived cohort of MS patients is summarized in **Figure 1A**. Performance of the MS classifying algorithms at 95% specificity is presented in **Table 1**. The algorithm that includes both codified and NLP-extracted narrative variables (**Figure S1, Table S3**) showed the best performance and accurately identified MS patients with an area under the curve (AUC) of 0.958±0.006 on a receiver-operator characteristics analysis (**Figure S2**). Setting the false positive rate or specificity at 95%, this combined algorithm had a sensitivity of 82.7±2.4%, positive predictive value (PPV) of 92.1±0.6% and negative predictive value (NPV) of 88.8±1.7%. The algorithm containing both codified and narrative variables exhibited superior performance (better sensitivity, PPV, NPV and accuracy) when compared with the other three versions of the MS classification algorithm: the model based only on the MS ICD-9 code (the prevailing approach), the codified data-only model and the NLP data-only model (**Table 1**).

**Table 1:** Performance of the four models of the EHR algorithm for identifying multiple sclerosis patients (at 95% specificity).

Model^a^	Sensitivity (SE)	PPV (SE)	NPV (SE)	AUC (SE)
**ICD**	0.600 (0.058)	0.894 (0.013)	0.769 (0.029)	0.890 (0.013)
**COD**	0.764 (0.038)	0.916 (0.007)	0.849 (0.023)	0.937 (0.010)
**NLP**	0.758 (0.034)	0.914 (0.006)	0.849 (0.021)	0.941 (0.008)
**ALL**	0.827 (0.024)	0.921 (0.006)	0.888 (0.017)	0.958 (0.006)

a The *ICD* model uses the number of ICD-9 code for MS as the only variable. The *Codified* (*COD*) model includes codified variables in addition to the number of ICD-9 code for MS. The *NLP* model includes narrative variables extracted from clinical texts. The combined (*ALL*) model uses both codified and narrative variables. Performance parameters are calculated using 0.632 bootstrap cross-validation in the training set. The standard errors are estimated based on 1,000 bootstrap replications.

Abbreviations: *AUC,* area under the curve; *NPV*, negative predictive value; *PPV*, positive predictive value; *SE,* standard error of the estimates.

We applied the combined algorithm to the pool of 22,610 patients with at least one MS-related ICD-9 code in the Partners Healthcare EHR system and identified 5,495 MS patients at 95% specificity as the EHR-based MS cohort for the remainder of the study (see **Table 2** for demographics). Consistent with the sensitivity of the algorithm, this MS virtual cohort captured 85.5% of the MS subjects who are registered in the Partners MS Center database.

**Table 2:** Characteristics of the EHR-derived cohort of multiple sclerosis (MS) patients and the subset of patients who receive care at a subspecialty MS Center^a^.

Parameter	EHR-derived Cohort (n = 5,495)	MS Center Subset (n = 4,241)
Sex (% female)	73%	73%
Race/Ethnicity (% non-Hispanic white)	72%	75%
Age at first ICD-9 code for MS, years (median [Q1–Q3])	41 [33–49]	40 [32–49]
Duration of follow-up, years (median [Q1–Q3])	8.4 [3.5–13.7]	9.1 [4.3–14.4]
Number of ICD-9 code for MS per patient (median [Q1–Q3])	22 [8–49]	26 [9–55]
Number of MRI brain per patient (median [Q1–Q3])	6 [3]–[12]	8 [4]–[14]
Number of MRI cervical spine per patient (median [Q1–Q3])	4 [2]–[6]	4 [2]–[7]
Number of entries by a MS neurologist per patient(median [Q1–Q3])	47 [13–124]	56 [12–139]
Number of prescriptions for MS disease modifyingtreatment per patient (median [Q1–Q3])	5 (2–13)	6 (3–15)
Receiving MS disease modifying treatment, %	49%	55%

a A subset of the patients in the EHR-derived MS cohort receives neurological care at the Partners MS Center where neuroimaging and clinical outcomes are available. For comparison, our cohort shares similar basic demographic characteristics as an independent MS patient registry from the North American Research Committee on Multiple Sclerosis (NARCOMS): 73% of the NARCOMS patients are female, 90% are self-described White, mean age at diagnosis is 37 years, and 52% of the patients are receiving immune modulatory therapy [32].

Abbreviation: ICD-9 = 340 is the diagnostic code for MS.

To address whether EHR data can be harnessed to provide clinically meaningful phenotypes, we studied a subset of the MS patients in the EHR-derived cohort who receive neurological care at an MS Center (“clinical cohort”). From the MS Center database, we obtained two types of MS disease course data that have been prospectively collected but are not available in routine medical records: (1) brain parenchymal fraction (BPF), a measure of whole brain volume derived from semi-automated segmentation of brain MRI scans with manual correction, (2) multiple sclerosis severity score (MSSS), a measure of disease severity and disability adjusted for disease duration.

### Deriving Whole Brain Volume from EHR Data

**Figure 2:**
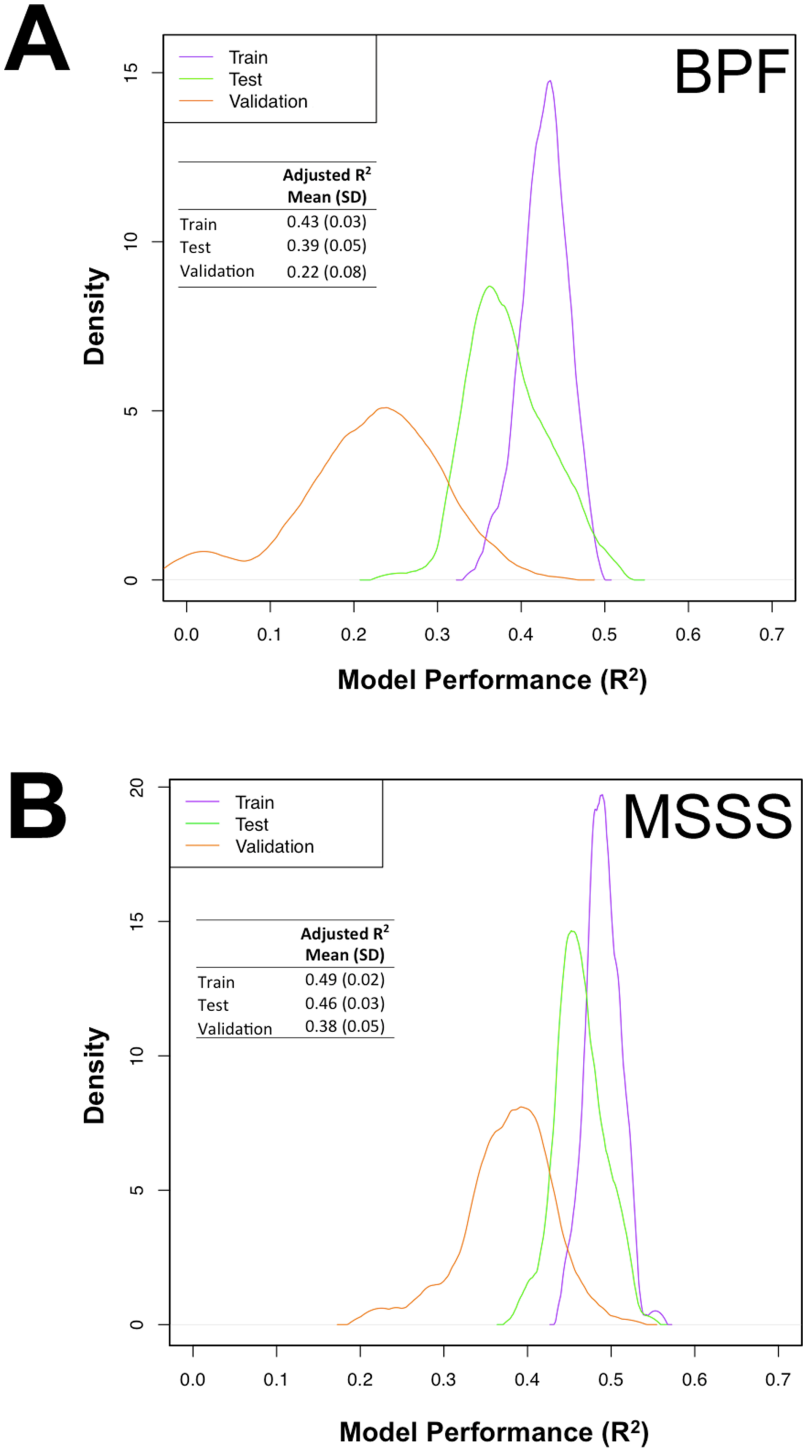
Density distribution of the performance (adjusted R^2^) of the EHR algorithm for deriving brain parenchymal fraction (A), and multiple sclerosis severity score (B). Performance is measured as variance that explains the correlation between the derived and true outcomes after adjusting for the number of variables in the model.

To derive a surrogate measure of whole brain volume in our MS patients, we used patients of European ancestry who have at least one measure of manually corrected BPF (n = 789) collected in the clinical cohort study. **Figure 1B** summarizes our double cross-validation approach to develop the BPF algorithm in the training and test sets. We then assessed its performance in the validation set. **Figure 2A** shows the correlation between the derived BPF and the true BPF in each set of subjects. As expected, the correlation (R^2^) is the best in the training set where it is over-fitted. The correlation in the validation set is the most accurate assessment of the algorithm’s performance as it is applied to an independent subset of subjects. We repeated this algorithm building process 100 times, permuting the assignment of subjects to each set and creating a distribution of R^2^ values for each set. The mean value for each set is reported. The BPF algorithm in the validation sets has an adjusted mean R^2^ of 0.22±0.08.

The algorithm for deriving BPF contains both codified and narrative EHR variables (**Figure S4A**, **Table S4**). In addition, the clinical cohort database provided the variables of age at symptom onset and disease duration at the time of MRI since these variables are known to correlate with brain volume [19], [30] but not available in routine medical records. The performance of the BPF algorithm in the validation set is reduced from a mean R^2^ of 0.22±0.08 to 0.01±0.04 when only codified variables are included and to 0.0007±0.09 when only narrative variables are included in the algorithm. Thus, neither type of EHR data alone is sufficient to produce an estimate of BPF. When the BPF algorithm includes only sex, age of symptom onset and disease duration, it has a mean R^2^ of 0.28±0.06, suggesting that the existing EHR variables are not informative for deriving a surrogate measure of BPF.

### Deriving MS Severity Score from EHR Data

We used the same approach to develop an EHR algorithm that derives a surrogate measure of MS severity in patients of European ancestry within the EHR-based cohort who have at least one Multiple Sclerosis Severity Score (MSSS, n = 1835) (**Figure 1B**). Following the double cross-validation approach, the correlation between the derived and true MSSS in the independent validation sets had an adjusted mean R^2^ of 0.38±0.05 (**Figure 2B**).

Because the algorithm for deriving MSSS also contains both codified and narrative variables from the EHR as well as sex and age of symptom onset from the clinical cohort database (**Figure S4B**, **Table S5**), we assessed the contribution of the different types of variables. (MSSS already accounts for disease duration.) The performance of the MSSS algorithm in the validation set was reduced from an adjusted mean R^2^ of 0.38±0.05 to 0.16±0.06 when only codified variables are included and to 0.31±0.06 when only narrative variables are included in the algorithm. Thus, the NLP-extracted narrative data are highly informative in the MSSS algorithm. When the MSSS algorithm included only sex and age of symptom onset, it has a mean R^2^ of 0.05±0.02, further confirming that the EHR variables are necessary for deriving MSSS.

### Distribution of EHR-derived MSSS in Relapsing-Remitting and Progressive MS Patients

To illustrate the validity of EHR-derived measures of MSSS, we tested whether we could reproduce the known differences in MSSS measures observed between relapsing-remitting and progressive MS patients. Based on published reports [31] and observations from our own Partners MS Center, progressive MS patients are known to have, on average, more disability than relapsing-remitting MS patients after adjusting for age and disease duration. Among the MS patients within the EHR-based cohort with both BPF and MSSS measures and known disease categories from the MS Center database at the time of the measures (n = 542), 59 are primary progressive or secondary progressive, and 483 are relapsing-remitting. We divided these patients into a discovery and replication set and compared the difference in observed and derived MSSS between progressive and relapsing-remitting patients in both sets (**Table 3**). After considering the differences in sex, age of symptom onset and disease duration, primary and secondary progressive MS patients have a higher mean EHR-derived MSSS than relapsing-remitting MS patients (discovery, p = 9.42×10^−23^; and replication, p = 1.56×10^−12^), consistent with observations based on actual MSSS (**Table 3**).

**Table 3:** EHR-derived MS severity score (MSSS) captures the difference between progressive MS and relapsing-remitting MS patients.

	Discovery Set^b^ (n = 329)			Validation Set^b^ (n = 213)		
Outcome^a^	PPMS/SPMS (n = 34) Mean (SE)	RRMS (n = 295) Mean (SE)	*p-value*	PPMS/SPMS(n = 25) Mean (SE)	RRMS (n = 188) Mean (SE)	*p-value*
Observed MSSS	3.86 (0.27)	0.86 (0.10)	1.55E-23	4.36 (0.29)	0.73 (0.11)	3.32E-26
Derived MSSS	2.90 (0.18)	0.98 (0.06)	9.42E-23	3.22 (0.18)	1.10 (0.07)	1.56E-12

a Observed MSSS is based on actual data from MS patients who receive care at the Partners MS Center. Derived MSSS is based on algorithm with 40% frequency cut-off for EHR variables.

b Patients with known MS disease category were divided into a discovery set (n = 329, including 34 PPMS/SPMS patients and 295 RRMS patients) and a validation set (n = 213, including 25 PPMS/SPMS and RRMS patients). For the observed measure of MSSS, ANOVA was performed and the comparison was adjusted for sex, age of symptom onset and disease duration as covariates. For derived surrogate measure of MSSS, t-test was performed. The effects of sex, age of symptom onset, and disease duration are accounted for in the derivation of the surrogate measure of MSSS.

Abbreviations: *BPF*, brain parenchymal fraction; *MSSS*, multiple sclerosis severity score; *PPMS*, primary progressive multiple sclerosis; *RRMS*, relapsing-remitting multiple sclerosis; *SPMS*, secondary progressive multiple sclerosis.

## Discussion

Using a medical informatics framework and rigorous statistical methodology, our study showcases an approach that begins to harness routine EHR data for accurate identification of patients with a complex neurologic disease and for deriving a highly relevant clinical outcome heretofore only available in research studies. Specifically, our study leverages the EHR data of a large cohort of MS patients to provide the Multiple Sclerosis Severity Score, an important indicator of disease severity that is not part of routine medical records. Although the derived MSSS measure is not yet robust for research, this approach provides the first steps towards harnessing existing EHR data for patient-oriented research in neurological diseases that will enable exploration of the many unique features of the EHR data as EHR systems become widely adopted across the health care landscape.

Our approach embraces the rich complexity of the EHR data. The incorporation of sophisticated codified data and NLP-extracted narrative data improved the performance of the EHR algorithm to identify MS patients when compared to the approach relying only on ICD-9 codes. With this approach, we established a cohort of 5,495 MS patients, including a subset that is part of a patient cohort based at MS Center. This unique “virtual cohort” enables analyses that integrate the new EHR-derived variables with traditional clinical research data. Further, we demonstrated that NLP-extracted narrative data are necessary for generating an informative estimate for MSSS. As a demonstration of its clinical relevance, EHR-derived MSSS captures the difference between the two main subgroups of MS patients: relapsing-remitting patients who generally recover neurological function after a relapse and progressive patients who experience decline in function. With future improvement in EHR data and informatics methods, we will enhance the MSSS algorithm (to reach at least R = 0.8) so that this surrogate measure may be potentially integrated into the EHR system to allow better monitor of patient outcomes and for research.

EHR data did not contribute meaningfully to the performance of the BPF algorithm, which can be almost entirely explained by variables obtained from the clinical cohort database: age of first symptom and disease duration. This illustrates the limitation that EHR variables considered here are not sufficient to inform every pertinent outcome measure. To provide a surrogate of brain volume, critical information to supplement EHR data can be obtained using questionnaires to ascertain age of symptom onset and disease duration. Thus, integration of EHR data and data from clinical research tools such as questionnaires provides a path for future investigations that leverage the strengths of both approaches. Brain volume is not routinely measured in clinical care, but it is correlated with disease course and is an important research measure in MS. Surrogate measures of brain volume derived from these combined approaches could enable the exploration of hypotheses that cannot be effectively investigated at smaller sample sizes, despite the use of more accurate measures. In the future, we plan to enhance the algorithm development for whole brain volume by applying automated feature selection methods to the entire narrative text based on the medical ontology systems such as SNOMED-CT instead of only expert-selected EHR variables. Further, disease duration may be derived if the date of the first neurological symptom can be captured by more sophisticated NLP capability.

Our study has two other limitations. First, our algorithms for MS were developed and tested within a single EHR system that links two major tertiary care hospitals and affiliates. We have not yet tested the portability of our algorithms. This is an important next step, as we will seek replication of the EHR algorithms for classifying MS and deriving MS disease outcomes in the EHR systems of other healthcare institutions. If proven portable, this approach promises efficient and cost-effective development of multi-center cohorts to address research questions highly relevant to neurological patients. It is reassuring that our group has developed a similar EHR algorithm for classifying rheumatoid arthritis and demonstrated its portability in two other academic medical centers with limited retraining of the algorithm [11].

The second limitation involves our current inability to finely dissect the temporal relationship between the EHR data and indicators of MS disease severity. Specifically, the EHR data used for algorithm development represent aggregate information as of the time of the MS data mart creation, and the latest available measures of BPF and MSSS from the MS Center clinical cohort do not necessarily occur after the aggregate information has been collected. Thus, our study demonstrated cross-sectional associations and should not be construed as predictive algorithms as this would imply that the EHR data occurred before the BPF or MSSS measures. As medical informatics technologies continue to improve the parsing of temporal relationships, truly predictive algorithms for brain volume and disease severity will emerge and be translated into the clinical arena to guide patient management.

In the age of personalized medicine, EHR data provide another complementary layer of biomedical data. The challenge is to integrate EHR data with other data to improve patient care. Our study in MS showcases an informatics approach that harnesses routine EHR data to derive MSSS, a well-accepted and clinically meaningful disease measure heretofore available only in research studies. If replicated, our novel informatics approach will enable the development of multi-center cohorts and facilitate testing of a variety of new hypotheses leveraging the unique features of the EHR data to address MS disease activity, comorbidities, treatment response and presymptomatic disease. These efforts also hold the promise of establishing automated monitors of an individual patient’s disease trajectory using EHR and aiding clinician’s task of delivering more individualized patient management. Finally, while MS was used as a proof of principle in this study, our approach has the potential of being applied in other complex neurological diseases.

## Supporting Information

Figure S1 **The final algorithm for identifying multiple sclerosis patients based on EHR data contains both codified variables and natural language processing (NLP)-extracted narrative variables.** To facilitate portability of the algorithm, the estimates of beta coefficient are not normalized.
(DOC)

Figure S2 **The receiver operator characteristic analysis of the EHR algorithm for identifying multiple sclerosis patients.**
(DOC)

Figure S3 **Relative frequency of the EHR variables for deriving brain parenchymal fraction (A), and multiple sclerosis severity score (B) in a subset of the EHR-derived multiple sclerosis cohort with observed data.**
(DOC)

Figure S4 **The final algorithm for deriving brain parenchymal fraction (A), and for deriving multiple sclerosis severity score (B), based on EHR variable frequency threshold at 40%.**
(DOC)

Table S1 **List of variables considered for developing brain parenchymal fraction (BPF) and MS disease severity (MSSS) algorithms.**
(DOC)

Table S2 **Association between actually observed and EHR-derived brain parenchymal fraction (BPF) and multiple sclerosis severity score (MSSS).**
(DOC)

Table S3 **List of codified and narrative variables in the final EHR algorithm for identifying multiple sclerosis patients.**
(DOC)

Table S4 **List of variables in the final EHR algorithm for brain parenchymal fraction (BPF).**
(DOC)

Table S5 **List of variables in the final EHR algorithm for MS disease severity (MSSS).**
(DOC)

Text S1 **Supplemental methods and results.**
(DOC)
