# Supplementary material for: Modeling Disease Severity in Multiple Sclerosis Using Electronic Health Records
Source: PLoS One. 2013 Nov 11;8(11):e78927. doi: 10.1371/journal.pone.0078927 (PMC3823928; doi:10.1371/journal.pone.0078927)

**Figure S1. The final algorithm for identifying multiple sclerosis patients based on EHR data contains both codified variables and natural language processing (NLP)-extracted narrative variables.** To facilitate portability of the algorithm, the estimates of beta coefficient are not normalized.

Abbreviation: *ICD9,* International Classification of Disease 9^th^ edition; *dmt*, any of the disease modifying treatment for multiple sclerosis; *edss*, Kurtzke Expanded Disability Status Scale.


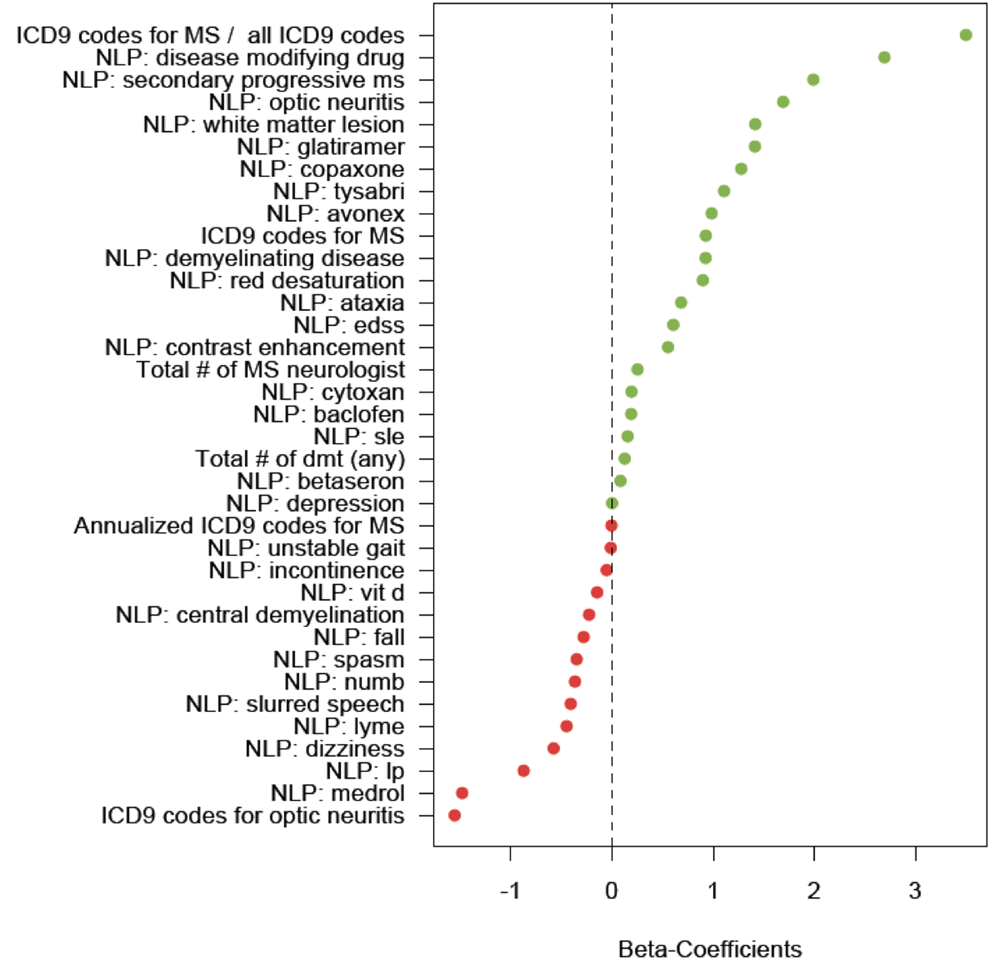

Supplement: Figure S1 — The final algorithm for identifying multiple sclerosis patients based on EHR data contains both codified variables and natural language processing (NLP)-extracted narrative variables. To facilitate portability of the algorithm, the estimates of beta coefficient are not normalized. (DOC) [file pone.0078927.s001.doc]
