# Supplementary material for: Modeling Disease Severity in Multiple Sclerosis Using Electronic Health Records
Source: PLoS One. 2013 Nov 11;8(11):e78927. doi: 10.1371/journal.pone.0078927 (PMC3823928; doi:10.1371/journal.pone.0078927)

**Figure S2. The receiver operator characteristic analysis of the EHR algorithm for identifying multiple sclerosis patients.**

Abbreviation: The *ICD* model uses the number of ICD-9 code for MS as the only variable. The *Codified* (*COD*) model includes codified variables in addition to the number of ICD-9 code for MS. The *NLP* model includes narrative variables extracted from clinical texts. The combined (*ALL*) model uses both codified and narrative variables.


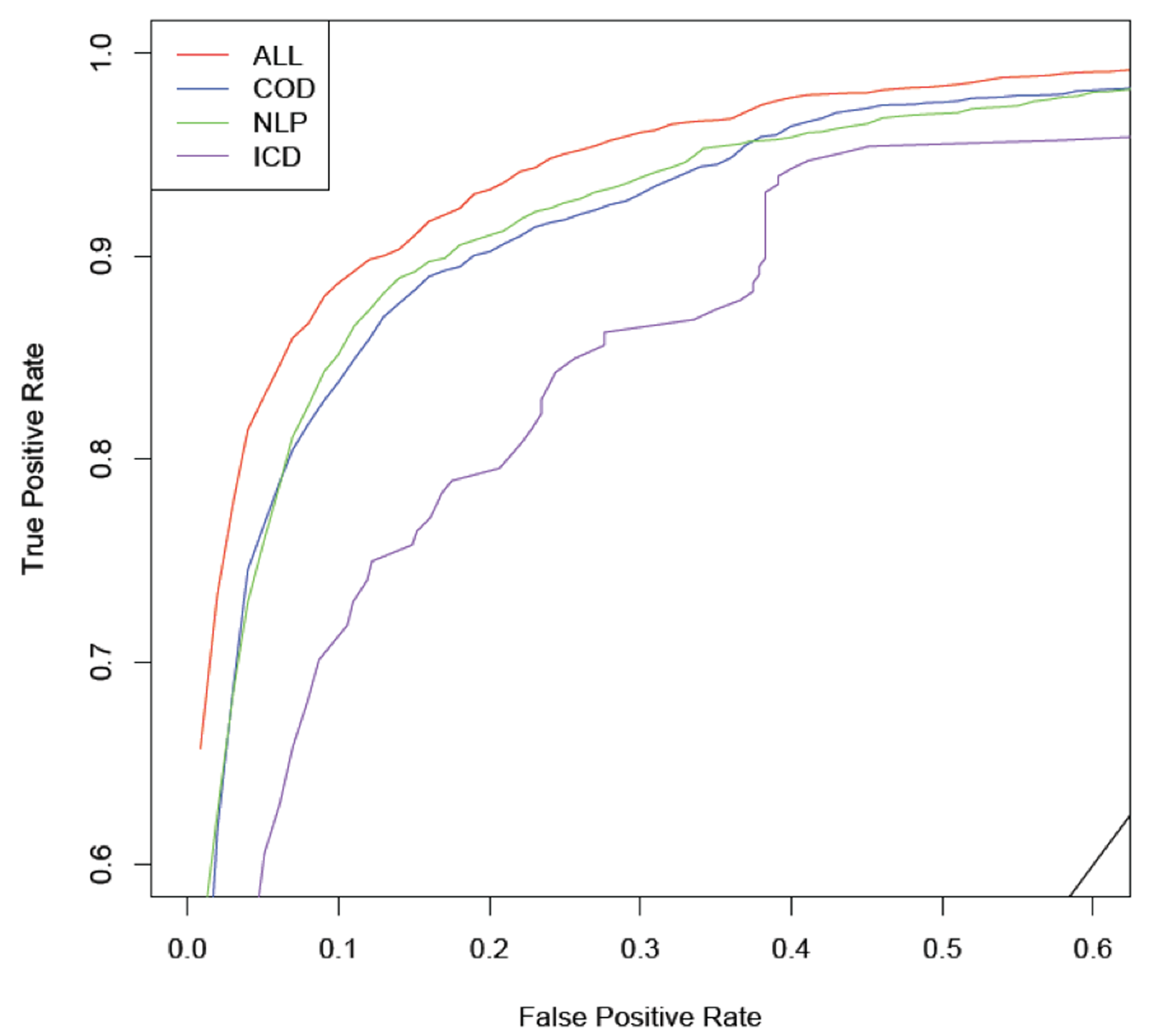

Supplement: Figure S2 — The receiver operator characteristic analysis of the EHR algorithm for identifying multiple sclerosis patients. (DOC) [file pone.0078927.s002.doc]
