# Supplementary material for: Modeling Disease Severity in Multiple Sclerosis Using Electronic Health Records
Source: PLoS One. 2013 Nov 11;8(11):e78927. doi: 10.1371/journal.pone.0078927 (PMC3823928; doi:10.1371/journal.pone.0078927)

**Figure S3. Relative frequency of the EHR variables for deriving brain parenchymal fraction (A), and multiple sclerosis severity score (B) in a subset of the EHR-derived multiple sclerosis cohort with observed data.**

Abbreviation: *AGE.FS*, age of the first MS-related neurological symptom; *BPF,* brain parenchymal fraction; *COD.dmt*, electronic prescriptions for any of the disease modifying treatment for MS; *COD.icd_340,* number of ICD-9 diagnostic code for MS (340); *COD.icd340_all,* proportion of total diagnostic codes that are MS, number of ICD-9 diagnostic code for MS (340) divided by the total number of any type of ICD-9 diagnostic code; *COD.icd340_annual,* annualized number of ICD-9 diagnostic code for MS (340); *COD.mri_bra,* number of brain MRI; *COD.mri_csp*, number of cervical spine MRI; *COD.ms_neu,* number of entries by a MS neurologist; *COD.opt_neu,* number of diagnostic code for optic neuritis; *COD.w340,* number of ICD-9 diagnostic code for MS (340) that are at least one week apart; *DD_fromFS,* disease duration from the first symptom; *edss,* Kurtzke Expanded Disability Status Scale; *ms,* multiple sclerosis; *MSSS,* multiple sclerosis severity score; *msex,* male sex; *NLP*, natural language processing extracted narrative variable; *NLP.LP*, lumbar puncture; *NLP.mri*, magnetic resonance imaging; *NLP.ocb,* oligoclonal band; *NLP.pml,* progressive multifocal leukoencephalopathy; *NLP.vep*, visual evoked potential; *NLP.vit.d*, Vitamin D.


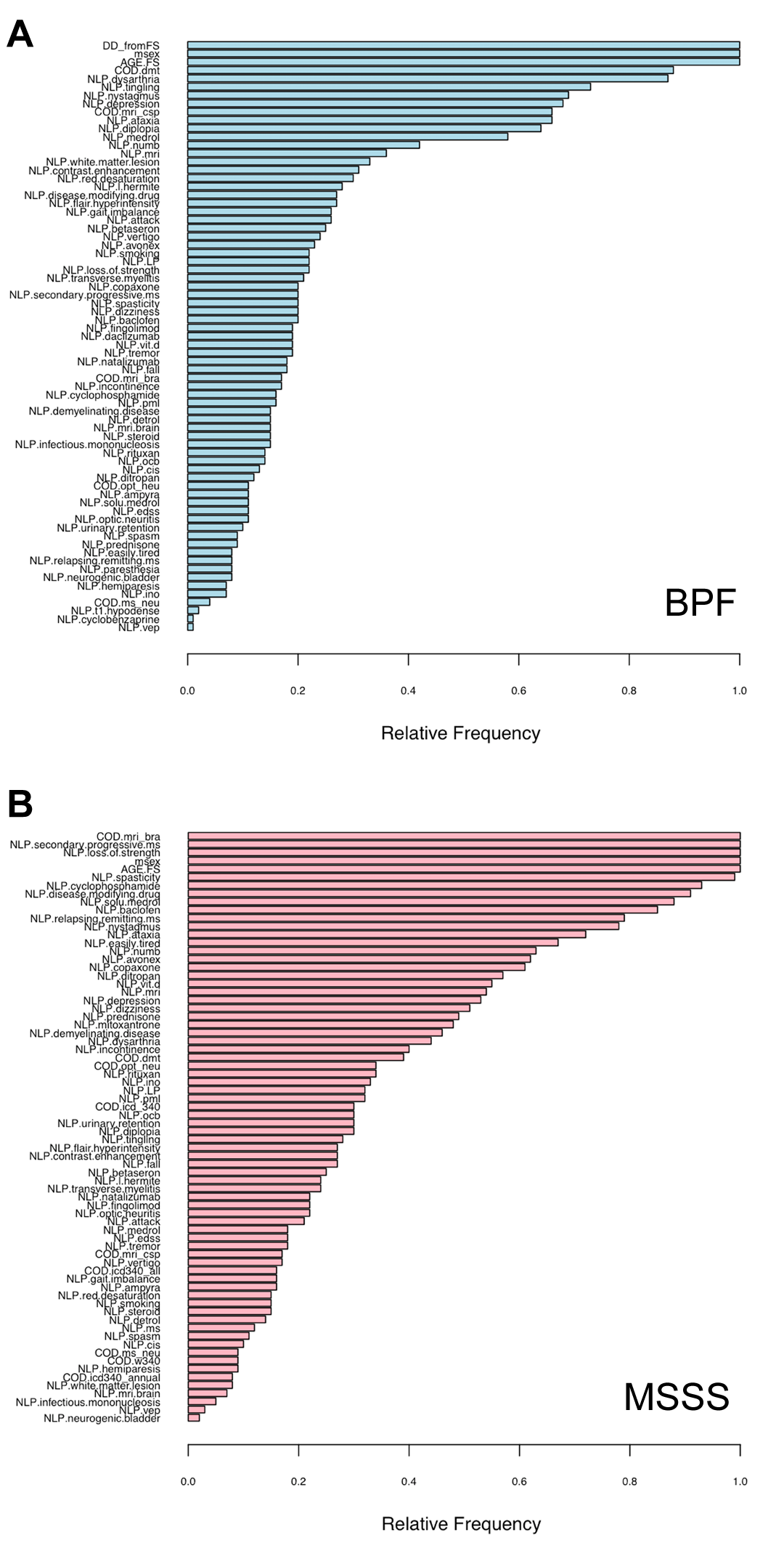

Supplement: Figure S3 — Relative frequency of the EHR variables for deriving brain parenchymal fraction (A), and multiple sclerosis severity score (B) in a subset of the EHR-derived multiple sclerosis cohort with observed data. (DOC) [file pone.0078927.s003.doc]
