# Supplementary material for: Modeling Disease Severity in Multiple Sclerosis Using Electronic Health Records
Source: PLoS One. 2013 Nov 11;8(11):e78927. doi: 10.1371/journal.pone.0078927 (PMC3823928; doi:10.1371/journal.pone.0078927)

**Figure S4. The final algorithm for deriving brain parenchymal fraction (A), and for deriving multiple sclerosis severity score (B), based on EHR variable frequency threshold at 40%.**

Abbreviation: *AGE.FS*, age of the first MS-related neurological symptom; *COD.dmt*, electronic prescriptions for any of the disease modifying treatment for MS; *COD.mri_bra,* number of brain MRI; *COD.mri_csp*, number of cervical spine MRI; *DD_fromFS,* disease duration from the first symptom; *msex,* male sex; *NLP.mri*, magnetic resonance imaging; *NLP.ocb,* oligoclonal band; *NLP.vit.d*, Vitamin D.


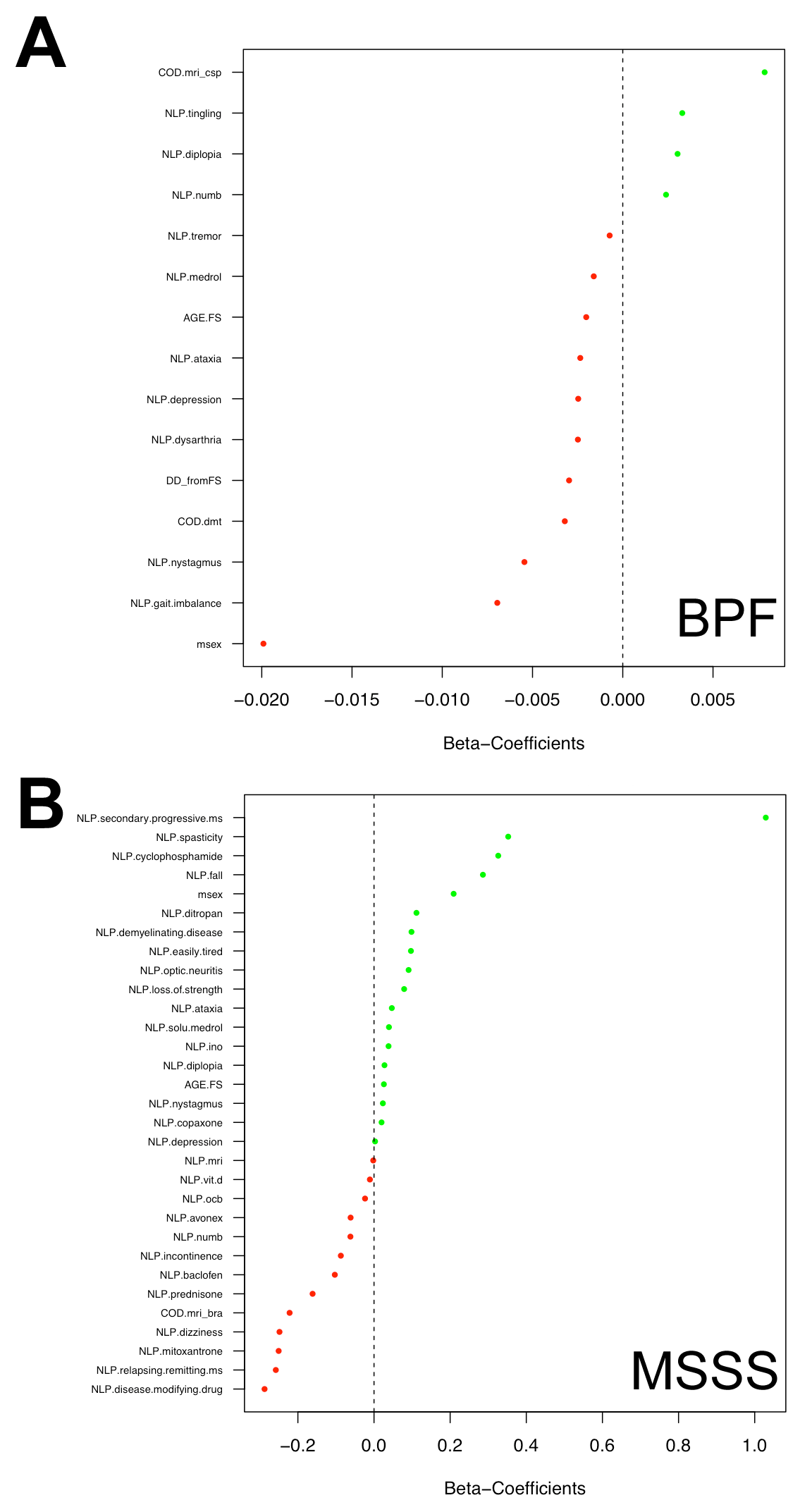

Supplement: Figure S4 — The final algorithm for deriving brain parenchymal fraction (A), and for deriving multiple sclerosis severity score (B), based on EHR variable frequency threshold at 40%. (DOC) [file pone.0078927.s004.doc]
