# Supplementary material for: Modeling Disease Severity in Multiple Sclerosis Using Electronic Health Records
Source: PLoS One. 2013 Nov 11;8(11):e78927. doi: 10.1371/journal.pone.0078927 (PMC3823928; doi:10.1371/journal.pone.0078927)
Supplement: Table S1 — List of variables considered for developing brain parenchymal fraction (BPF) and MS disease severity (MSSS) algorithms. (DOC) [file pone.0078927.s005.doc]

**Table S1.** List of variables considered for developing brain parenchymal fraction (BPF) and MS disease severity (MSSS) algorithms

| **Variables Considered** | **Explanation ^b^** |
| --- | --- |
| AGE.FS ^a^ | Age of first neurological symptom |
| msex | Sex, male |
| DD_fromFS (not for MSSS) | Disease duration from first symptom |
| NLP^a^.abatacept |  |
| NLP.acth | “adrenocorticotropic hormone” |
| NLP.acute.disseminated.encephalomyelitis |  |
| NLP.aggressive.ms | “aggressive multiple sclerosis” |
| NLP.alemtuzumab |  |
| NLP.alfuzosin |  |
| NLP.aminopyridine |  |
| NLP.ampyra |  |
| NLP.ataxia |  |
| NLP.attack |  |
| NLP.avonex |  |
| NLP.baclofen |  |
| NLP.baep | “brainstem auditory evoked potential” |
| NLP.balo.concentric.sclerosis |  |
| NLP.benign.ms | “benign multiple sclerosis” |
| NLP.betaseron |  |
| NLP.black.hole |  |
| NLP.brain.fog |  |
| NLP.cdms | “clinically definitive multiple sclerosis” |
| NLP.cerebral.atrophy |  |
| NLP.chronic.microangiopathic.change |  |
| NLP.chronic.microvascular.ischemic.change |  |
| NLP.cis | “clinically isolated syndrome” |
| NLP.cladribine |  |
| NLP.cognitive.impairment |  |
| NLP.contrast.enhancement |  |
| NLP.copaxone |  |
| NLP.cyclobenzaprine |  |
| NLP.cyclophosphamide |  |
| NLP.daclizumab |  |
| NLP.demyelinating.disease |  |
| NLP.depression |  |
| NLP.detrol |  |
| NLP.devic.disease |  |
| NLP.diplopia |  |
| **Variables Considered** | **Explanation** |
| NLP.disease.modifying.drug |  |
| NLP.dissemination.in.space |  |
| NLP.dissemination.in.time |  |
| NLP.ditropan |  |
| NLP.dizziness |  |
| NLP.dysarthria |  |
| NLP.dyssomnias |  |
| NLP.easily.tired |  |
| NLP.edss | “Kurtzke Expanded Disability Status Scale” |
| NLP.fall |  |
| NLP.fingolimod |  |
| NLP.flair.hyperintensity | “Fluid attenuated inversion recovery (FLAIR) hyperintensity” |
| NLP.flavoxate |  |
| NLP.gait.imbalance |  |
| NLP.gray.matter.atrophy |  |
| NLP.hemiparesis |  |
| NLP.hemiplegia |  |
| NLP.ifn.beta-1a | “interferon beta-1a” |
| NLP.inability.to.perform.activities.of.daily.living |  |
| NLP.incontinence |  |
| NLP.infarct |  |
| NLP.infectious.mononucleosis |  |
| NLP.ino | “internuclear ophthalmoplegia” |
| NLP.juxtacortical.lesion |  |
| NLP.l'hermite |  |
| NLP.leukoaraiosis |  |
| NLP.leukodystrophy |  |
| NLP.lipohyalinosis |  |
| NLP.loss.of.strength |  |
| NLP.LP | “lumbar puncture” |
| NLP.malignant.ms |  |
| NLP.marburg.multiple.sclerosis |  |
| NLP.medrol |  |
| NLP.mitoxantrone |  |
| NLP.mri | “magnetic resonance imaging” |
| NLP.mri.brain | “magnetic resonance imaging of brain” |
| NLP.ms | “multiple sclerosis” |
| NLP.multifocal.signal.abnormality |  |
| NLP.natalizumab |  |
| NLP.neurogenic.bladder |  |
| **Variables Considered** | **Explanation** |
| NLP.numb | “numb” or “numbness” |
| NLP.nystagmus |  |
| NLP.ocb | “oligoclonal band(s)” |
| NLP.optic.neuritis |  |
| NLP.ovoid |  |
| NLP.paresthesia |  |
| NLP.perventricular.white.matter |  |
| NLP.physical.disability |  |
| NLP.pml | “progressive multifocal leukoencephalopathy” |
| NLP.poor.vision |  |
| NLP.ppms | “primary progressive multiple sclerosis” |
| NLP.prednisone |  |
| NLP.prms | “progressive relapsing multiple sclerosis” |
| NLP.radiologically.isolated.syndrome |  |
| NLP.red.desaturation |  |
| NLP.relapse |  |
| NLP.relapsing.remitting.ms | “relapsing-remitting multiple sclerosis” |
| NLP.rituxan |  |
| NLP.sanctura |  |
| NLP.secondary.progressive.ms | “secondary progressive multiple sclerosis” |
| NLP.sensory.deficit |  |
| NLP.sexual.dysfunction |  |
| NLP.shilder.disease |  |
| NLP.sildenafil |  |
| NLP.small.vessel.infarcts |  |
| NLP.smoking |  |
| NLP.solu-medrol |  |
| NLP.somatosensory.evoked.potential |  |
| NLP.spasm |  |
| NLP.spasticity |  |
| NLP.spinal.cord.atrophy |  |
| NLP.steroid |  |
| NLP.subcortical.lesion |  |
| NLP.t1.hyperdense |  |
| NLP.t1.hypodense |  |
| NLP.tingling |  |
| NLP.transverse.myelitis |  |
| NLP.tremor |  |
| NLP.trigeminal.neuralgia |  |
| NLP.tumefactive.ms |  |
| **Variables Considered** | **Explanation** |
| NLP.uhthoff's.phenomenon |  |
| NLP.uric.acid |  |
| NLP.urinary.retention |  |
| NLP.vep | “visual evoked potential” |
| NLP.vertigo |  |
| NLP.vision.loss |  |
| NLP.vit.d | “vitamin D” |
| NLP.well-circumscribed.lesion |  |
| NLP.wheel.chair.bound |  |
| NLP.white.matter.lesion |  |
| COD^a^.dmt | Electronic prescriptions for any of the disease modifying treatment for MS |
| COD.icd_340 | Number of ICD-9 diagnostic code for MS (340) |
| COD.icd340_all | Proportion of total diagnostic codes that are MS: number of ICD-9 diagnostic code for MS (340) divided by the total number of any type of ICD-9 diagnostic code |
| COD.icd340_annual | Annualized number of ICD-9 diagnostic code for MS (340) |
| COD.mri_bra | Number of brain MRI |
| COD.mri_csp | Number of cervical spine MRI |
| COD.ms_neu | Number of entries by a MS neurologist |
| COD.opt_neu | Number of diagnostic code for optic neuritis |
| COD.dmt | Prescriptions for any of the disease modifying treatment for MS (alemtuzumab, daclizumab, fingolimod, glatiramer, interferon beta-1a, interferon beta-1b, mitoxantrone, natalizumab, rituximab) |

^a^ Abbreviation: *COD,* codified variable; *NLP*, natural language processing extracted narrative variable

^b^ Explanation is provided where there is an acronym in the variable name. NLP captures words or phrases. For narrative variables obtained using NLP, each word or phrase (e.g., “numb”) is mapped to a concept unique identifier (CUI) using a comprehensive medical terminology system, SNOMED CT. Multiple words can be mapped to the same CUI. For example, “numb” and “numbness” are mapped to the same CUI (C0028643), whereas “paresthesia” uses a different CUI (C0030554). For simplicity, not all words are included in the variable name (column A). Our NLP method simply counts the sum of the number of positive and negative occurrences of each word or phrase in a patient’s entire medical record. The NLP method does not capture the timing, location or duration of a word (e.g., “numb”).
