# Supplementary material for: Modeling Disease Severity in Multiple Sclerosis Using Electronic Health Records
Source: PLoS One. 2013 Nov 11;8(11):e78927. doi: 10.1371/journal.pone.0078927 (PMC3823928; doi:10.1371/journal.pone.0078927)
Supplement: Table S2 — Association between actually observed and EHR-derived brain parenchymal fraction (BPF) and multiple sclerosis severity score (MSSS). (DOC) [file pone.0078927.s006.doc]

**Table S2.** Association between actually observed and EHR-derived brain parenchymal fraction (BPF) and multiple sclerosis severity score (MSSS)

|  | | **Training Set** (n=361) | | | **Test Set** (n=240) | | |
| --- | --- | --- | --- | --- | --- | --- | --- |
| **Outcome** | **Number of EHR Variables** | **R^2^** | **Estimate** | ***p-Value*** | **R^2^** | **Estimate** | ***p-Value*** |
|  |  |  |  |  |  |  |  |
| ***Observed MSSS ^a^*** |  |  |  |  |  |  |  |
| **Observed BPF** |  | 0.08 | -9.47 | 5.28E-08 | 0.05 | -8.69 | 0.0006 |
| **Derived BPF (30%)** | 17 | 0.11 | -16.72 | 2.30E-10 | 0.05 | -12.10 | 0.0003 |
| **Derived BPF (40%)** | 13 | 0.11 | -17.23 | 1.26E-10 | 0.07 | -14.21 | 4.44E-05 |
| **Derived BPF (50%)** | 12 | 0.11 | -17.04 | 2.37E-10 | 0.06 | -13.79 | 8.91E-05 |
|  |  |  |  |  |  |  |  |
| ***Observed BPF ^a^*** |  |  |  |  |  |  |  |
| **Observed MSSS** |  | 0.08 | -0.01 | 5.28E-08 | 0.05 | -0.01 | 0.0006 |
| **Derived MSSS (30%)** | 37 | 0.15 | -0.02 | 2.98E-14 | 0.14 | -0.02 | 1.50E-09 |
| **Derived MSSS (40%)** | 27 | 0.15 | -0.02 | 4.74E-14 | 0.14 | -0.02 | 3.11E-09 |
| **Derived MSSS (50%)** | 22 | 0.15 | -0.02 | 5.44E-14 | 0.14 | -0.02 | 1.62E-09 |
|  |  |  |  |  |  |  |  |

^a^ Observed BPF and MSSS are based on actual data from MS patients that are collected in the Partners MS Center database. Derived BPF and MSSS are developed from algorithms with the frequency cut-off for EHR variables at 30%, 40% or 50%.
