# Supplementary material for: Modeling Disease Severity in Multiple Sclerosis Using Electronic Health Records
Source: PLoS One. 2013 Nov 11;8(11):e78927. doi: 10.1371/journal.pone.0078927 (PMC3823928; doi:10.1371/journal.pone.0078927)
Supplement: Table S3 — List of codified and narrative variables in the final EHR algorithm for identifying multiple sclerosis patients. (DOC) [file pone.0078927.s007.doc]

**Table S3.** List of codified and narrative variables in the final EHR algorithm for identifying multiple sclerosis patients

| **Variables** | **Beta Coefficient or Estimate** | **Standard Error** |
| --- | --- | --- |
| (Intercept) | -3.470 | 0.712 |
| COD.icd340_all | 3.500 | 1.196 |
| NLP.disease modifying drug | 2.694 | 2.325 |
| NLP.secondary progressive ms | 1.991 | 1.895 |
| NLP.optic neuritis | 1.692 | 0.849 |
| NLP.white matter lesion | 1.416 | 0.946 |
| NLP.glatiramer | 1.413 | 1.963 |
| NLP.copaxone | 1.278 | 0.549 |
| NLP.tysabri | 1.108 | 1.194 |
| NLP.avonex | 0.985 | 0.546 |
| COD.icd_340 | 0.927 | 0.927 |
| NLP.demyelinating disease | 0.925 | 0.693 |
| NLP.red desaturation | 0.898 | 1.013 |
| NLP.ataxia | 0.683 | 0.509 |
| NLP.edss | 0.606 | 0.989 |
| NLP.contrast enhancement | 0.554 | 0.563 |
| COD.ms_neu | 0.253 | 0.226 |
| NLP.cytoxan | 0.193 | 0.453 |
| NLP.baclofen | 0.189 | 0.364 |
| NLP.sle | 0.155 | 0.878 |
| COD.dmt | 0.126 | 0.497 |
| NLP.betaseron | 0.084 | 0.648 |
| NLP.depression | 0.001 | 0.263 |
| COD.icd340_annual | -0.005 | 0.027 |
| NLP.unstable gait | -0.011 | 1.150 |
| NLP.incontinence | -0.053 | 0.379 |
| NLP.vit d | -0.148 | 0.292 |
| NLP.central demyelination | -0.227 | 0.659 |
| NLP.fall | -0.281 | 0.450 |
| NLP.spasm | -0.349 | 0.603 |
| NLP.numb | -0.366 | 0.392 |
| NLP.slurred speech | -0.408 | 1.102 |
| NLP.lyme | -0.449 | 0.579 |
| NLP.dizziness | -0.578 | 0.431 |
| NLP.lp | -0.874 | 0.615 |
| NLP.medrol | -1.482 | 1.519 |
| COD.opt_neu | -1.556 | 2.029 |

Please see Table S1 and its table legend for explanation of the variables.
