# Supplementary material for: Modeling Disease Severity in Multiple Sclerosis Using Electronic Health Records
Source: PLoS One. 2013 Nov 11;8(11):e78927. doi: 10.1371/journal.pone.0078927 (PMC3823928; doi:10.1371/journal.pone.0078927)
Supplement: Table S4 — List of variables in the final EHR algorithm for brain parenchymal fraction (BPF). (DOC) [file pone.0078927.s008.doc]

**Table S4.** List of variables in the final EHR algorithm for brain parenchymal fraction (BPF)

| **Variables** | **Beta Coefficient or Estimate** | **Standard Error** |
| --- | --- | --- |
| (Intercept) | 0.9304 | 0.0105 |
| COD.mri_csp | 0.0079 | 0.0036 |
| NLP.tingling | 0.0033 | 0.0023 |
| NLP.diplopia | 0.0030 | 0.0018 |
| NLP.numb | 0.0024 | 0.0020 |
| NLP.tremor | -0.0007 | 0.0020 |
| NLP.medrol | -0.0016 | 0.0017 |
| AGE.FS | -0.0020 | 0.0002 |
| NLP.ataxia | -0.0024 | 0.0021 |
| NLP.depression | -0.0025 | 0.0017 |
| NLP.dysarthria | -0.0025 | 0.0022 |
| DD_fromFS | -0.0030 | 0.0003 |
| COD.dmt | -0.0032 | 0.0017 |
| NLP.nystagmus | -0.0054 | 0.0017 |
| NLP.gait.imbalance | -0.0070 | 0.0038 |
| msex | -0.0199 | 0.0050 |

Please see Table S1 and its table legend for explanation of the variables.
