# Supplementary material for: Modeling Disease Severity in Multiple Sclerosis Using Electronic Health Records
Source: PLoS One. 2013 Nov 11;8(11):e78927. doi: 10.1371/journal.pone.0078927 (PMC3823928; doi:10.1371/journal.pone.0078927)
Supplement: Table S5 — List of variables in the final EHR algorithm for MS disease severity (MSSS). (DOC) [file pone.0078927.s009.doc]

**Table S5.** List of variables in the final EHR algorithm for MS disease severity (MSSS)

| **Variables** | **Beta Coefficient or Estimate** | **Standard Error** |
| --- | --- | --- |
| (Intercept) | 0.7344 | 0.4112 |
| NLP.secondary.progressive.ms | 1.0291 | 0.1408 |
| NLP.spasticity | 0.3524 | 0.0787 |
| NLP.cyclophosphamide | 0.3263 | 0.0738 |
| NLP.fall | 0.2860 | 0.0968 |
| msex | 0.2092 | 0.1647 |
| NLP.ditropan | 0.1115 | 0.1235 |
| NLP.demyelinating.disease | 0.0984 | 0.0942 |
| NLP.easily.tired | 0.0970 | 0.0727 |
| NLP.optic.neuritis | 0.0908 | 0.0681 |
| NLP.loss.of.strength | 0.0791 | 0.0383 |
| NLP.ataxia | 0.0467 | 0.0734 |
| NLP.solu.medrol | 0.0392 | 0.0736 |
| NLP.ino | 0.0382 | 0.1359 |
| NLP.diplopia | 0.0275 | 0.0643 |
| AGE.FS | 0.0259 | 0.0067 |
| NLP.nystagmus | 0.0233 | 0.0586 |
| NLP.copaxone | 0.0197 | 0.0509 |
| NLP.depression | 0.0027 | 0.0628 |
| NLP.mri | -0.0021 | 0.1181 |
| NLP.vit.d | -0.0109 | 0.0751 |
| NLP.ocb | -0.0236 | 0.1050 |
| NLP.avonex | -0.0615 | 0.0490 |
| NLP.numb | -0.0620 | 0.0645 |
| NLP.incontinence | -0.0872 | 0.0785 |
| NLP.baclofen | -0.1030 | 0.0854 |
| NLP.prednisone | -0.1614 | 0.1095 |
| COD.mri_bra | -0.2218 | 0.1384 |
| NLP.dizziness | -0.2483 | 0.0814 |
| NLP.mitoxantrone | -0.2505 | 0.2494 |
| NLP.relapsing.remitting.ms | -0.2580 | 0.0982 |
| NLP.disease.modifying.drug | -0.2876 | 0.1364 |

Please see Table S1 and its table legend for explanation of the variables.
