# Supplementary material for: Modeling Disease Severity in Multiple Sclerosis Using Electronic Health Records
Source: PLoS One. 2013 Nov 11;8(11):e78927. doi: 10.1371/journal.pone.0078927 (PMC3823928; doi:10.1371/journal.pone.0078927)
Supplement: Text S1 — Supplemental methods and results. (DOC) [file pone.0078927.s010.doc]

**Supplemental Text**

***Establishing an EHR-based Cohort of MS Patients***

To develop a highly sensitive and specific EHR-based algorithm to classify patients with a high probability of MS, we first created an “MS data mart” containing the complete medical records of all patients from the Partners HealthCare EHR with at least one MS-related ICD-9 code (n=22,610). 595 patients were then randomly selected for a training set to develop the algorithm. Manual review of medical records by two neurologists confirmed 251 cases of definitive MS (42.3%) in the training set. The remaining patients in the training set: (1) have a diagnosis of *probable* MS without a neurologist or MRI confirmation (9.4%), (2) have a diagnosis of clinically isolated syndrome or precursor to MS (5.4%), (3) do not have a diagnosis of MS (39.5%), or (4) lack sufficient information in the EHR to determine a diagnosis (3.3%).

To select informative features to include in an algorithm and estimate their regression coefficients to classify MS, we fit an adaptive LASSO penalized logistic regression with tuning parameter selected via the Bayesian Information Criteria. From an expert-defined list of EHR variables (14 codified and 275 NLP-extracted narrative), this approach selected 36 EHR features that are informative for classifying MS: 6 codified and 30 narrative variables (**Figure S1**), of which the variable with the highest beta-coefficient is a codified term enumerating the proportion of all ICD-9 codes that are MS, followed by the narrative term “disease modifying drug”. To facilitate the portability of the algorithm, beta-coefficient estimates of the variables are shown as non-normalized values. Using manually verified MS diagnosis and a bootstrap cross-validation, we assessed the performance of the different models of the classifying algorithm in the training set: (1) the *ICD9* model, which uses the number of ICD-9 code for MS as the only variable, (2) the *Codified* (*COD*) model includes codified variables in addition to ICD-9 code for MS, (3) the *Natural Language Processing* (*NLP*) model includes only narrative variables extracted from clinical texts, and (4) the combined (*ALL*) model using both codified and narrative data (**Table 1**). Setting the false positive rate or specificity at 95%, this combined model comprised of both codified data and NLP-extracted narrative data showed the best performance with a true positive rate or sensitivity of 83%, positive predictive value (PPV) of 92% and negative predictive value (NPV) of 89% and area under the curve (AUC) of 0.96 (**Figure S2**).

The combined model of the MS classifying algorithm was then applied to all 22,610 patients with at least one MS-related ICD-9 code in the MS data mart. In total, the algorithm captured 5,495 MS patients at 95% specificity. For the remainder of the study, we used the algorithm comprised of both codified and narrative data at 95% specificity to define the EHR-based MS cohort (see **Table 2** for demographics). A review of 100 randomly selected patients from this EHR-based MS cohort by two neurologists showed 93% accuracy in MS diagnosis with an inter-reviewer agreement of 96%, consistent with the PPV of the algorithm. Where there was a discrepancy between the two reviewers regarding MS diagnosis, the senior neurologist repeated the chart review and cast the final decision.

Further, the EHR-based MS cohort is linked to a biobank of discarded blood samples from routine clinical care known as Crimson. This system enables the efficient collection of plasma samples and buffy-coated tubes from which DNA can be extracted. We anticipate collecting approximately 1,650 unique blood samples in a 12-month period.

***EHR Algorithms for Deriving Whole Brain Volume and Disease Severity in MS***

To determine the optimal frequency threshold for the EHR variables, we examined whether the algorithm-derived BPF and MSSS display the same modest correlation as the true BPF and MSSS. For this analysis, we used a subset of the subjects with both actual BPF and MSSS data, and divided them into a training set (60%) and test set (40%). In the training set, we fitted algorithms to obtain the magnitude of the beta-coefficients using EHR variables that made it into the algorithms at least 30%, 40% or 50% of the time from the 5-fold cross-validation stepwise regression. In the test set, we applied those coefficients as weights to calculate an EHR-derived BPF and MSSS for each subject. We then assessed the association between BPF and MSSS using R^2^. Specifically, we measured the association between (1) observed BPF and MSSS, (2) EHR-derived BPF and observed MSSS, (3) observed BPF and EHR-derived MSSS, (4) EHR-derived BPF and MSSS.

The EHR data used for developing the BPF and MSSS algorithms consisted of expert-defined codified and narrative variables extracted using NLP (**Table S1**). Given that the variable selection was repeated 100 times in the algorithm building process, we report the frequency of selection of each variable by the algorithm (**Figure S3**). We observed that the inclusion of additional variables does not improve the algorithm performance beyond a threshold. To determine the optimal variable frequency cut-off for inclusion in the final BPF and MSSS algorithms, we compared results at different frequency thresholds. Specifically, we assessed whether the BPF and MSSS algorithms replicate the known modest correlation between brain atrophy and disease severity in MS. This modest correlation between actual BPF and MSSS is shown in the clinical cohort subjects (R^2^=0.04). We assessed the performance of the algorithms containing EHR variables at different frequency thresholds in MS patients of European ancestry with observed BPF and MSSS measures (n=654) (**Table S2**). Using the observed MSSS, the correlation with EHR-derived BPF in the test set (as measured by R^2^) improves with increasing variable frequency until the 40% threshold. Beyond 40%, the performance of the BPF algorithm begins to decline. Using the observed BPF, the correlation of the EHR-derived MSSS in the test set similarly improves with increasing variable frequency until the 40% threshold, but the algorithm performed equally well at the 30% and 50% thresholds (**Table S2**). Overall, we selected 40% as the frequency threshold for variables in the cohort for deriving BPF and MSSS. **Figure S4** shows the codified and narrative variables (at 40% frequency) that are selected for the BPF and MSSS algorithms. To facilitate the portability of the algorithm, beta-coefficient estimates of the variables are shown as non-normalized values. In the BPF algorithm, the variable with the highest beta-coefficient is a codified term enumerating the total number of cervical spine MRI scans, followed by the narrative term “tingling” as the second highest. In the MSSS algorithm, the variables with the three highest beta-coefficients are all narrative terms (“secondary progressive MS”, “spasticity”, “cyclophosphamide”).

Because we manually added three non-EHR-derived variables from the Partners MS Center database into the algorithm for deriving BPF (i.e., age at first symptom, disease duration of MS, and sex), we examined the contribution of these added variables on the performance of the algorithm in the independent validation subgroup. The performance (mean R^2^) of the algorithm for deriving BPF in the validation set is significantly reduced from 0.22 ± 0.08 (**Figure 2A**) to 0.08 ± 0.08 when repeating the feature selection process without forcing in these three variables, and to -0.01 ± 0.10 when specifically excluding these variables. Because we found that the age at first symptom was on average 5.4 years less than age at first ICD-9 code for MS (p<0.001), the age of the first ICD-9 code for MS is not the best substitute for age of the first symptom or to impute disease duration.

Because we manually added two non-EHR-derived variable from the Partners MS Center database into the algorithm for deriving MSSS (i.e., age at first symptom and sex), we examined the contribution of these variables on the performance of the algorithm in the independent validation subgroup. The performance (mean R^2^) of the algorithm for deriving MSSS in the independent validation set is minimally reduced from 0.38 ± 0.05 (**Figure 2B**) to 0.34 ± 0.05 when specifically excluding these variables.

**References for Supplemental Text**

1. Murphy S, Churchill S, Bry L, Chueh H, Weiss S, et al. (2009) Instrumenting the health care enterprise for discovery research in the genomic era. Genome Res 19: 1675-1681.

2. De Stefano N, Matthews PM, Filippi M, Agosta F, De Luca M, et al. (2003) Evidence of early cortical atrophy in MS: relevance to white matter changes and disability. Neurology 60: 1157-1162.

3. Sanfilipo MP, Benedict RH, Sharma J, Weinstock-Guttman B, Bakshi R (2005) The relationship between whole brain volume and disability in multiple sclerosis: a comparison of normalized gray vs. white matter with misclassification correction. NeuroImage 26: 1068-1077.
